# Interplay Between Lipid Metabolism and Autophagy

**DOI:** 10.3389/fcell.2020.00431

**Published:** 2020-06-03

**Authors:** Yangchun Xie, Jingbo Li, Rui Kang, Daolin Tang

**Affiliations:** ^1^Department of Oncology, The Second Xiangya Hospital, Central South University, Changsha, China; ^2^Department of Surgery, UT Southwestern Medical Center, Dallas, TX, United States

**Keywords:** autophagy, lipid, metabolism, lipophagy, ferritinophagy, clockophagy, mitophagy, disease

## Abstract

Autophagy is a self-eating process of using lysosomes to degrade macromolecular substances (e.g., proteins and organelles) that are damaged, degenerated, or aging. Lipid metabolism is the synthesis and degradation of lipids (e.g., triglycerides, steroids, and phospholipids) to generate energy or produce the structural components of cell membranes. There is a complex interplay between lipid metabolism (e.g., digestion, absorption, catabolism, biosynthesis, and peroxidation) and autophagy machinery, leading to the modulation of cell homeostasis, including cell survival and death. In particular, lipid metabolism is involved in the formation of autophagic membrane structures (e.g., phagophores and autophagosomes) during stress. Moreover, autophagy, especially selective autophagy (e.g., lipophagy, ferritinophagy, clockophagy, and mitophagy), promotes lipid catabolism or lipid peroxidation-induced ferroptosis through the degradation of various substances within the cell. A better understanding of the mechanisms of autophagy and possible links to lipid metabolism will undoubtedly promote potential treatments for a variety of diseases.

## Introduction

The morphological changes of macroautophagy were first observed using electron micrographs of rat liver after perfusion with glucagon for 4 h by Thomas Ashford and Keith Porter in 1962 (Ashford and Porter, 1962). Later, Christian de Duve coined the term “autophagy” from the ancient Greek language to describe the process of “self-eating” (Klionsky, 2008). It is now known that macroautophagy is one of the lysosome-mediated degradation pathways that plays a critical role in maintaining homeostasis (Yang and Klionsky, 2010). In general, increased macroautophagy can promote cell survival in response to various stresses, such as starvation, radiation, hypoxia, and oxidative stress. Macroautophagy can remove injured organelles, unused proteins, or invading microorganisms for normal cell activity and metabolism during aging, differentiation, or infection (Mizushima, 2007; Kaur and Debnath, 2015). However, deficient, excessive, or dysfunctional macroautophagy is implicated in various human diseases and pathologic conditions (Levine and Kroemer, 2019).

Lipids are one of the important nutrients of the body, providing it with energy and essential fatty acids (FAs) or their derivatives. There are three types of lipids, namely triglycerides (TGs), steroids, and phospholipids (Fahy et al., 2005, 2009). TGs have a chemical name of triacylglycerols (TAGs), built from one glycerol molecule and three FAs. Steroids include hormones and cholesterol. Notably, cholesterol, the most abundant steroid lipid in the body, also plays a role in the production of hormones. Phospholipids form double-layered membranes with water-soluble molecules on the outside of the cell membrane and water-insoluble molecules in the inside (DeBose-Boyd, 2018). The levels of lipids are controlled by lipid metabolism, which is a complex process involved in the biosynthesis and degradation of lipids. The first step of lipid metabolism is hydrolysis. As hydrophobic molecules, lipids need to be solubilized to produce free FAs (FFAs) and monoacylglycerol (MAG) (Mu and Porsgaard, 2005) through enzymatic hydrolysis in the digestive system. The second step involves the absorption, packaging, and transporting of the FAs from the digestive system into the rest of the body (Ko et al., 2020). TGs, also known as fats, are mainly obtained from daily food. Lipogenesis is the process of synthesizing TGs, mostly completed in the liver. Dysfunction in the storage or breakdown of lipids can cause cell dysfunction, even cell death (Zechner et al., 2012).

Recent years have seen a rapid growth in the study of the interplay between macroautophagy and lipid metabolism (Liu and Czaja, 2013; Caron et al., 2015; Jaishy and Abel, 2016; Thelen and Zoncu, 2017). In this review, we introduce the basic process of macroautophagy and summarize recent progress in understanding the impact of lipid metabolism on macroautophagy.

## Overview of Autophagy

**FIGURE 1:**
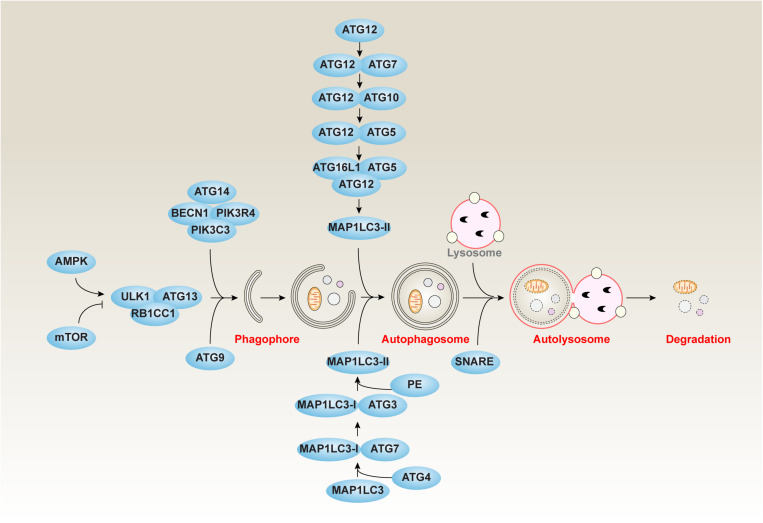
The core molecular machinery of autophagy. Autophagy is a dynamic process involving the formation of several specific membrane structures, such as phagophores, autophagosomes, and autolysosomes. The ATG proteins associated with other regulators play a complex role in the autophagic process of induction, nucleation, elongation, fusion, and degradation.

Autophagy can be divided into three main types, namely chaperone-mediated autophagy (CMA), microautophagy, and macroautophagy, according to the transporting manners of cell materials into lysosomes (Dikic and Elazar, 2018). CMA is mediated by heat shock proteins that bind the target substrates to deliver them to lysosomes for degradation (Majeski and Dice, 2004). During microautophagy, long-lived proteins can be directly engulfed by lysosomal membrane to degrade in lysosomes (Li et al., 2012). Macroautophagy (hereafter autophagy) is a well-studied dynamic process, which is involved in the formation of several specific membrane structures, such as phagophores, autophagosomes, and autolysosomes (Dikic and Elazar, 2018) (Figure 1). The phagophores, also known as the isolation membranes, can engulf and isolate the cytoplasmic components to produce subsequent autophagosomes, a double membrane structure. The autophagosome further fuses with the lysosome to yield autolysosomes, leading to the degradation of the sequestered cytosolic material via the lysosome hydrolases.

At the molecular level, the formation of membrane structures of autophagy is controlled by autophagy-related (ATG) genes, which are conserved genes from yeast to humans (Levine and Kroemer, 2019). The ATG-coded proteins can form different complexes that are regulated by their posttranslational modifications (Xie et al., 2015). The ATG proteins associated with other regulators play a complex role in the autophagic process of induction, nucleation, elongation, fusion, and degradation (Dikic and Elazar, 2018).

### Induction

Autophagy is initiated by the formation of a phagophore that originates in the membranes of Golgi apparatus, endoplasmic reticulum (ER), endosome, mitochondria, or the plasma membrane. The induction of autophagy is controlled by the unc-51–like autophagy-activating kinase 1 (ULK1, a homolog of Atg1 in yeast) kinase complex, including the core component of ULK1, ATG13, and RB1 inducible coiled-coil 1 (RB1CC1, also known as FIP200). In addition to ULK1, ULK2 may have similar function in autophagy induction. Notably, two upstream kinases, namely mammalian target of rapamycin complex 1 (mTORC1) and AMP-activated protein kinase (AMPK), can inhibit or promote, respectively, the ULK1 kinase complex in response to environmental stresses (Holczer et al., 2019).

### Nucleation

The class III phosphatidylinositol 3-kinase (PtdIns3K) complex, mainly containing phosphatidylinositol 3-kinase, catalytic subunit type 3 (PIK3C3)/VPS34, BECN1 (also known as Atg6 in yeast), and ATG14 (also known as beclin-1-associated autophagy-related key regulator [Barkor] or ATG14L), plays a key role in the nucleation of phagophores (McKnight and Zhenyu, 2013). One of the key functions of the PtdIns3K complex is the generation of phosphatidylinositol-3-phosphate (PtdIns3P), a phosphoinositide that serves as a landmark on the membrane to recruit other factors involved in the process of autophagosome formation (Bernard and Klionsky, 2014). BECN1 is a multifunctional protein that not only promotes autophagy, but also controls cellular sensitivity to regulated cell death, such as apoptosis and necroptosis, through its binding partners (Kang et al., 2011). ATG14 plays an important role in the formation of autophagosomes (Zhong et al., 2009). ATG14 acts as a specific targeting factor for PI3KC3 to autophagosome membranes to maintain membrane curvature (Fan et al., 2011). In addition, ATG14 blocks connexins-mediated inhibitory effect on autophagy during autophagasome formation (Bejarano et al., 2014).

### Elongation

Subsequent to nucleation, the phagophore expands by membrane addition, which is accomplished by 2 ubiquitin-like (Ubl) conjugation systems, the ATG12-ATG5 conjugation system and the microtubule-associated protein 1 light chain 3 (MAP1LC3/LC3) conjugation system (Ohsumi, 2001). The ATG12-ATG5 conjugate can further bind ATG16L1 (also known as Atg16 in yeast) to form a ATG12-ATG5-ATG16L1 complex at phagophores. MAP1LC3 exhibits two forms, namely MAP1LC3-I and MAP1LC3-II. At baseline, most MAP1LC3 is MAP1LC3-I. In contrast, the production of MAP1LC3-II is increased in response to autophagic stimulus that is essential for the formation of the autophagosome and subsequent degradation of cargos through the binding to autophagy receptors, such as sequestosome 1 (SQSTM1, also known as p62) and calcium-binding and coiled-coil domain 2 (CALCOCO2, also known as NDP52). In addition to Ubl conjugation systems, ATG9-mediated cycling systems contribute to the elongation of the phagophore. ATG9 is thought to move from the *trans*-Golgi network or late endosomes to the phagophore and is regulated by the activity of ULK1, PtdIns3K, and mitogen-activated protein kinase 14 (MAPK14, also known as p38) (Young et al., 2006; Webber and Tooze, 2010). In addition to MAP1LC3, other orthologs of yeast Atg8, such as GABA type A receptor-associated protein (GABARAP) and GABA type A receptor-associated protein-like 2 (GABARAPL2, also known as GATE-16), also contribute to autophagosome formation in some cases (Schaaf et al., 2016).

### Fusion and Degradation

Once autophagosome formation is complete, the outer membrane of the autophagosome fuses to lysosomes to produce autolysosome, and the cellular materials (e.g., mitochondria and ER) and invading pathogens are destroyed by enzymes in lysosomes (Pankiv et al., 2007). Although many factors affect the fusion between autophagosome and lysosome, the soluble *N*-ethylmaleimide–sensitive factor attachment protein receptor (SNARE) family seems to play a key role in the formation of autolysosomes (Nakamura and Yoshimori, 2017). In addition, ATG14 binds and stabilizes the SNARE complex, thereby promoting autophagosome-lysosomal fusion (Diao et al., 2015). The autophagosome marker MAP1LC3-II protein can be finally degraded with cargos or autophagy receptors through lysosomes (Mizushima and Yoshimori, 2007; Pankiv et al., 2007). Thus, autophagic flux is an important factor in monitoring the formation and degradation of autophagosomes (Klionsky et al., 2016; Yoshii and Mizushima, 2017). The cell membrane, one of resources of the phagophore, can be eventually digested by lysosomes or self-decomposed through autolysosome formation.

## Lipid Digestion and Autophagy

The digestion of lipids takes place mainly in the small intestine. As pre-digested (orally- and stomach-digested) food enters the small intestine, the lipids in the food are emulsified, thereby promoting the release of FAs from TAGs, and other lipids (e.g., phospholipids and cholesterol) are also dispersed in the small colloidal particles containing water and oil that are called mixed micelles. Emulsification increases the surface area between enzymes and lipids, thereby increasing the lipolytic effect of lipase. These enzymes include pancreatic lipase, colipase, cholesterol esterase, and phospholipase A2 (PLA2). The emulsified FAs are further catalyzed by the pancreatic lipase, the phospholipids by the PLA2, and the cholesterol ester by the cholesterol ester enzyme. As a result, the lipids in the food produce glycerides, FAs, cholesterol, and phospholipids, which significantly increases the solubilization of the mixed micelles (Ko et al., 2020).

Although the autophagy-lysosomal system is not directly involved in the digestion of intestinal lipids, it plays a central role in cellular food degradation (also known as intracellular digestion) (McVeigh et al., 2006). Digestion produces the biosynthetic precursors needed to regenerate partially disrupted structures, thereby generating the energy necessary for anabolic processes. Some core components of autophagy machinery have lipid kinase modulation activity, such as PIK3C3/VPS34 and BECN1, which are required to initiate autophagy during fasting (Pozuelo-Rubio, 2012; McKnight and Zhenyu, 2013). Consequently, this would affect the rate of energy maintenance upon acute starvation.

## Lipid Absorption and Autophagy

In the small intestine, mixed micelles containing FAs, glycerol, cholesterol, and phospholipids are transported to intestinal epithelial cells for absorption. The uptake and absorption of glycerol and FAs are affected by chain length. Short-chain FAs (≤12 C) can be directly absorbed into the blood by binding to albumin. Long-chain FAs (>12 C) and other lipids need to be transported across cell membranes through the action of transporters. Inside the cell, they will be resynthesized into TAGs in the ER and then transported into the Golgi apparatus, where they combine with cholesterol, phospholipids, and apolipoproteins to form a lipoprotein called chylomicrons protein. Lipoproteins are transporters that are responsible for transport from the origin to the destination through the blood and lymph. The solubility of lipoproteins in the bloodstream is due to the coating of apolipoprotein (Ko et al., 2020).

As mentioned above, intestinal epithelial cells are absorption cells of the small intestine and mediate the absorption of fats in the diet by secreting TAGs into the circulation. Generally, TAGs are stored in cytoplasmic lipid droplets (LDs) and are sequentially hydrolyzed for secretion according to changes in fat levels. The transfer and hydrolysis of TAG-containing LDs degraded by lysosomes are mediated by autophagy, a process called lipophagy (Singh et al., 2009). Therefore, LDs act as lipid reservoirs in the anabolic pathway, while lysosomes are dedicated to the degradation of intracellular components (Dugail, 2014). Diacylglycerol *O*-acyltransferase-1 (DGAT1) synthesizes TAG and is necessary for dietary fat absorption and storage. Recent studies have found a unique intestinal phenotype, abnormal TAG accumulation, and intestinal epithelial LD mobilization in DGAT1-deficient mice, resulting in delayed fat absorption and resistance to diet-induced obesity (Hung and Buhman, 2019). A high-fat diet results in increased lipid intake and intestinal fat deposition in yellow catfish, which adversely affects their lipid absorption. The underlying mechanism is that a high-fat diet upregulates lipogenesis, lipolysis, and FA transport, and it induces ER stress and activates autophagy. These effects on fat-induced changes in intestinal lipid uptake play an important regulatory role in the model of yellow catfish (Ling et al., 2019).

## Lipid Catabolism and Autophagy

Triglycerides and phospholipids are first broken down by lipase or phospholipase, respectively, which results in the release of FA chains from the glycerol carbon backbone. Glycerol can be phosphorylated to glycerol-3-phosphate and then converted to glyceraldehyde 3-phosphate by glycolysis. The released FAs are catabolized in a process called β-oxidation, which in turn removes two carbon acetyl groups from the end of the FA chain, thereby reducing NAD^++^ and FAD to produce NADH and FADH2, respectively. Electrons generated during β-oxidation can be used to make ATP through oxidative phosphorylation (Adeva-Andany et al., 2019). The acetyl groups produced during β-oxidation are carried into the Krebs cycle by coenzyme A, which causes them to degrade to CO_2_, generate ATP through substrate-level phosphorylation, and generate additional NADH and FADH2 molecules (Adeva-Andany et al., 2019).

The catabolism of stored lipids in LDs is related to a variety of metabolic pathways that provide molecules used to generate energy, membrane building blocks, and lipid signaling (Wang, 2015). Generally, autophagy is induced for cell survival during LD degradation, which is controlled by multiple molecules (Cabodevilla et al., 2013; Parray and Yun, 2017). In particular, lipophagy-mediated LD degradation via patatin-like phospholipase domain-containing 2 (PNPLA2, also known as ATGL) can release FFAs under starvation conditions. FFA produced by LD catabolism is either transported to mitochondria for β-oxidation, or converted back to LDs. The biogenesis of LDs under starvation is mediated by autophagy degradation of membrane organelles, and DGAT1 is required as an adaptive cytoprotective mechanism against lipotoxicity (Li et al., 2017). PNPLA2-mediated signaling through sirtuin 1 (SIRT1) is necessary and sufficient to induce lipophagy for subsequent LD catabolism and FA oxidation in hepatocytes (Sathyanarayan et al., 2017). The overexpression of perilipin 2 (PLIN2, also known as adipophilin), one of the most abundantly expressed LD proteins, protects LD from autophagy-dependent degradation, while its deficiency stimulates TG catabolism through autophagy, protecting mice against fatty liver diseases (Tsai et al., 2017). Sphingosine kinase 2 (SPHK2) is also required for the autophagy-mediated catabolism of intracellular LDs to prevent the development of atherosclerosis by reducing sphingosine content in macrophages (Ishimaru et al., 2019). Thyroid hormones induce FA β-oxidation through autophagy, which is associated with an increased delivery of FAs into mitochondria. Blockage of autophagy significantly reduces thyroid hormone-mediated FA β-oxidation *in vitro* and *in vivo* (Sinha et al., 2012).

Autophagy-mediated lipid catabolism can be regulated by transcription factors. The upregulation of transcription factor forkhead homeobox protein O1 (FOXO1) or lysosomal acid lipase (LIPA) increase autophagy-dependent LD degradation and subsequent FA release through AMPK-dependent β-oxidation in adipocytes upon nutrient restriction (Lettieri Barbato et al., 2013). Another transcriptional mechanism that links autophagy to lipid catabolism is the activation of transcription factor EB (TFEB) during starvation (Li et al., 2016; Napolitano and Ballabio, 2016). TFEB-mediated transcriptional induction of peroxisome proliferator-activated receptor gamma coactivator 1-alpha (PPARGC1A) and peroxisome proliferator-activated receptor alpha (PPARA) serves as a prosurvival response to nutrition deprivation (Settembre et al., 2013). Moreover, PPARA-induced TFEB activation or microRNA-33–mediated TFEB inhibition may form a feedback loop to further regulate lipid catabolism and FA β-oxidation (Ouimet et al., 2016, 2017; Kim et al., 2017). This process is also implicated in the response to ethanol-induced liver injury in mice (Thomes et al., 2019).

In addition to transcription factors, phosphoinositide-3-kinase regulatory subunit 4 (PIK3R4, also known as VPS15 in yeast) is critical for regulating PPARA activation. The loss of PIK3R4 inhibits autophagy and lipid catabolism through the accumulation of PPARA repressors, such as histone deacetylase 3 (HDAC3) and nuclear receptor corepressor 1 (NCOR1) (Iershov et al., 2019). CCAAT enhancer-binding protein alpha (CEBPA) also plays an essential role in promoting cell survival and FA β-oxidation during liver injury (Lu et al., 2015), although the mechanism remains unclear. Moreover, the activation of the small guanosine triphosphatase (GTPase) family (e.g., Rab7 and Rab18), BCL2 family (e.g., BIF1), or methionine metabolism plays a context-dependent role in the regulation of FA β-oxidation during autophagy (Schroeder et al., 2015; Liu et al., 2016; Zubiete-Franco et al., 2016; Bekbulat et al., 2019).

In yeast, LDs can also be turned over in vacuoles/lysosomes by microlipophagy, a process morphologically similar to microautophagy (van Zutphen et al., 2014). Microlipophagy is different from lipophagy and does not involve core autophagy proteins, but requires ESCRT components and newly identified VPS proteins (Vevea et al., 2015; Oku et al., 2017). Microlipophagy-dependent LDs depletion is triggered by AMPK activation, but not glucose starvation, amino acid deprivation or rapamycin treatment (Seo et al., 2017). In contrast, mTOR (Rahman et al., 2018), amino acid (Hatakeyama et al., 2019), and glucose (Iwama and Ohsumi, 2019) are important regulators of microautophagy.

CMA deficiency can cause lipid accumulation (Qiao et al., 2020), and vice versa, a high-fat diet and excessive cholesterol intake can inhibit CMA (Rodriguez-Navarro et al., 2012). Lysosome-associated membrane protein type 2A (LAMP2A) is a key protein in the CMA pathway. The accelerated degradation of LAMP2A determines the loss of lysosomal membrane stability. Nutrient deprivation is also an activator of CMA, which selectively degrades PLIN (e.g., PLIN2 and PLIN3) and promotes the hydrolysis of LDs (Kaushik and Cuervo, 2015). These findings support the role of CMA in lipid metabolism, but the precise molecular pathway remains unclear.

The dysfunction of autophagy-dependent lipid catabolism is implicated in several pathologic conditions. Thiodigalactoside plays a role in browning and lipid catabolism by jointly inhibiting GAL1 and ATG5, so it may have potential therapeutic significance for regulating energy homeostasis through its role in white adipose tissue (Parray and Yun, 2017). Autophagy-mediated lipid catabolism is activated as a compensation for glutaminolysis inhibition, which regulates tumor cell survival (Halama et al., 2018). Enteric infection can initiate the metabolic reprogramming of enterocytes toward lipid catabolism, which is controlled by ULK1-dependent lipophagy and the subsequent activation of dual oxidase 1 (DUOX1), a member of the NADPH oxidase family (Lee et al., 2018). These findings indicate a complex interplay between lipid catabolism and autophagy.

## Lipid Biosynthesis and Autophagy

### Fatty Acid Biosynthesis

**FIGURE 2:**
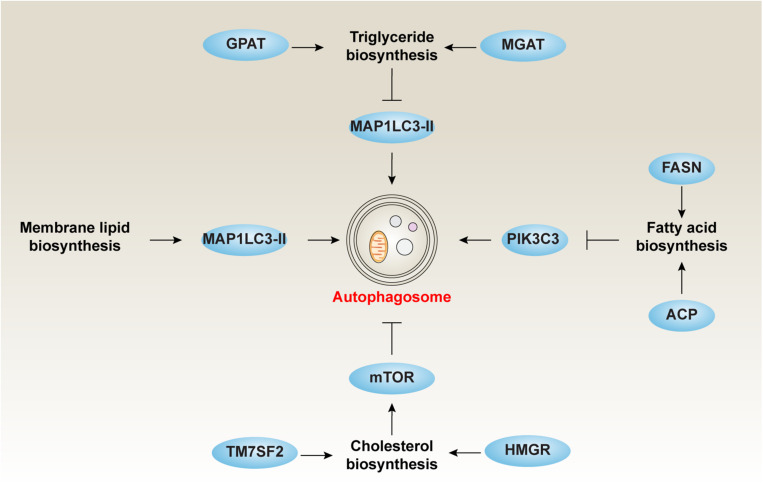
The role of lipid biosynthesis in autophagy. Inhibiting TG, CE, and FA or increasing membrane lipid biosynthesis is responsible for the induction of autophagy.

Fatty acids can be saturated (like palmitic acid and stearic acid) or unsaturated (like oleic acid). FAs are synthesized by gradually adding two-carbon units in the form of acetyl-CoA (Herman and Zhang, 2016). Acetyl-CoA is an important intermediate produced by the decarboxylation of pyruvate in the glucose breakdown pathway. However, the two-carbon units are produced not only by acetyl-CoA directly, but also by a carboxylated product of acetyl-CoA or malonyl-CoA. This process is catalyzed by acetyl-CoA carboxylase (Herman and Zhang, 2016). Moreover, the synthesis of FAs from acetyl-CoA or malonyl-CoA is mediated by fatty acid synthase (FASN) (Chirala and Wakil, 2004). Acyl carrier protein (ACP), a component of the FASN complex, is the core activator for FA biosynthesis (Herman and Zhang, 2016). The acyl groups get anchored to the CoA group of ACP through a thioester linkage. In many cases, inhibition of FA synthesis promotes autophagy (Figure 2).

Autophagy has been shown to regulate food intake and energy balance in hypothalamic agouti-related peptide (AgRP) neurons partly through the modulation of FA biosynthesis (Kaushik et al., 2011). The levels of AgRP, a neuropeptide produced in the brain by the AgRP neuron, is regulated by starvation-induced autophagy and subsequently the production of FFAs (Kaushik et al., 2011). In contrast, an autophagy deficiency in the hypothalamus may produce a lean body phenotype due to the lack of FFA-dependent AgRP production (Kaushik et al., 2011). The inhibition of autophagy by constitutive mTOR activity makes hypoxic cells dependent on exogenous desaturated lipids because that the level of unsaturated FA synthesized is reduced under hypoxia (Young et al., 2013). An increase in *de novo* synthesis of lipids is thought to be a metabolic adaptation of cancer cells, which can promote survival and metastasis. Increased FASN expression in colorectal cancer cells is associated with the inactivation of autophagy, including increased expression of SQSTM1 (Zaytseva et al., 2015). LD-deprived cells fail to induce autophagy due to accelerated lipid synthesis (Regnacq et al., 2016). In contrast, the administration of cerulenin (a potent inhibitor of FASN) or palmitic acid can restore nitrogen starvation-induced autophagy in the absence of LDs (Regnacq et al., 2016). It is worth noting that arachidonic acid, a long-chain polyunsaturated fatty acid (PUFA), is the main synthetic product under nitrogen deprivation, whereas monounsaturated oleic acid is the main product under phosphorous deprivation (Kokabi et al., 2019). The inhibition of PI3K signaling is responsible for lipogenesis rather than lipid hydrolysis by initiating *de novo* FA biosynthesis (Ramanan et al., 2018). These findings reveal a complex connection linking FA biosynthesis, nutrition status, and autophagy.

### Triglyceride Biosynthesis

There are three main ways for TGs to biosynthesize, namely the glycerol-3-phosphate (G3P) pathway [e.g., glycerol-3-phosphate acyltransferase (GPAT)], the dihydroxyacetone phosphate (DHAP) pathway, and the monoacylglycerol pathway [e.g., monoacylglycerol acyltransferase (MGAT)]. The G3P pathway, referred to as the Kennedy pathway, was identified by Eugene Kennedy in 1960, which is responsible for 90% of TG synthesis (Chai et al., 2017). Except in the intestine and adipocytes, TG synthesis begins with G3P (Chai et al., 2017). Glycerol is first phosphorylated by glycerin kinase, and then activated FA (e.g., fatty acyl-CoA) is used as a substrate for the addition of FA to produce phosphatidic acid. The phosphate group is then removed and the last FA is added.

Autophagy is implicated in the metabolic balance of liver TG. A lack of protein in the diet reduces the expression of autophagy receptor SQSTM1, increases the expression of autophagosome marker (MAP1LC3-II) as well as ER stress marker (the spliced isoform of XBP1), which helps accumulate TG in the liver (Yokota et al., 2016). Other regulators also participate in TG metabolism via modulating autophagy activity. For example, the loss of PLIN2 inhibits lipogenesis, reduces TG synthesis, and enhances autophagy (Irungbam et al., 2020). These findings suggest that TG metabolism plays a vital role in the modulation of autophagy (Figure 2).

### Cholesterol Biosynthesis

The biosynthesis of cholesterol generally takes place in the ER of hepatic cells and begins with acetyl-CoA, which is mainly derived from an oxidation reaction in the mitochondria (Alphonse and Jones, 2016). Acetyl-CoA is converted to 3-hydroxy- 3-methylglutaryl-CoA (HMG-CoA) by HMG-CoA synthase. HMG-CoA is then converted to mevalonate by HMG-CoA reductase (HMGR). This reaction is completed with the aid of NADPH, a co-factor for all reduction reactions during cholesterol synthesis (Jiang et al., 2018). Mevalonate can undergo a series of phosphorylations or decarboxylations to produce isoprenoid and isopentenyl pyrophosphate (Liao et al., 2016). A squalene synthase-mediated condensing reaction leads to the production of squalene. The first of the sterols is formed following the production of squalene and lanosterol. The conversion of lanosterol to cholesterol requires additional multiple biochemistry reaction steps (Cerqueira et al., 2016). Notably, the conversion of HMG-CoA to mevalonate by HMG-CoA reductase is the rate-limiting step of cholesterol biosynthesis, which is under strict regulatory control (Cerqueira et al., 2016). Consequently, HMGR has been long-recognized as a drug target to reduce serum cholesterol levels.

It is becoming increasingly clear that the inhibition of cholesterol synthesis is responsible for the induction of prosurvival autophagy through blocking the AKT-mTOR pathway in human blood cancer cells (Vilimanovich et al., 2015) (Figure 2). This process can be selectively attenuated by either mevalonate or squalene, but not by isopentenyl pyrophosphate (Vilimanovich et al., 2015). The depletion of transmembrane 7 superfamily member 2 (TM7SF2), a key regulator of cholesterol biosynthesis, results in the increased expression of FA catabolic enzymes accompanied by decreased lipid accumulation, autophagy, and tissue injury in mice exposed to endotoxin (Gatticchi et al., 2015). In addition, *de novo* sphingolipid biosynthesis is essential for autophagy induction in macrophages, which plays a protective role by clearing excess lipids from LDs through the turnover of ORMDL sphingolipid biosynthesis regulator 1 (ORMDL1) protein, a negative regulator of serine palmitoyl-CoA transferase activity (Wang et al., 2015b). Thus, the modulation of autophagy may influence cholesterol biosynthesis to reduce high-cholesterol–related diseases, such as atherosclerosis, heart disease, and stroke.

### Membrane Lipid Biosynthesis

Membrane lipids are necessary to form the structure of biological membranes (such as cell membranes and intracellular membranes) and are mainly composed of phospholipids, glycolipids, and sterols (e.g., cholesterol). They can be arranged in double layers together with intact and peripheral membrane proteins. Biosynthesis of membrane lipids involves the production of major membrane lipids and their transport from the site of synthesis into the cell membrane (van Meer et al., 2008).

It is thought that isolated membranes observed during autophagy are mainly derived from pre-formed organelle membranes (e.g., ER). Instead, the phagophore membrane expands along with localized phospholipid synthesis (Schutter et al., 2020). The original separation membrane is formed on ER from locally synthesized lipids, then an increase in the biosynthesis of the bilayer-forming phospholipids [phosphatidylcholine (PC), phosphatidylethanolamine (PE), and phosphatidylserine (PS)] occurs simultaneously with the induction of autophagy (Figure 2). PE conjugates the cytosolic MAP1LC3-I to form MAP1LC3-II, which is an important event in isolated membrane. The effect of PI3K on ER phosphatidylinositol coincides with the biogenesis of phospholipids. The two processes work together to help extend and assemble autophagosome particles (Girardi et al., 2011). The first step in *de novo* phospholipid synthesis at the ER is to make stable contact with nascent autophagosomes, which is essential for autophagy induction. Recent studies have shown that the conserved acyl-CoA synthetase FAA1 accumulates on nucleated phagophores, which is required for FA-mediated phospholipid synthesis and for promoting the assembly of phospholipids into autophagic membranes during phagophore elongation (Schutter et al., 2020). Glycosphingolipid is a key component of the eukaryotic cell membrane and is necessary for cavernous-mediated endocytosis and the function of glycosphingolipid-binding toxins (Sillence, 2007). Glycosphingolipid biosynthesis is restricted by enhanced autophagy, while its catabolism increases (Ghidoni et al., 1996). *De novo* sphingolipid biosynthesis is essential for autophagy induction (Wang et al., 2015a). Administering inhibitors to the first step of sphingolipid synthesis reduces autophagic activity by affecting autophagosome formation rather than the pre-structure formation of autophagosomes (Yamagata et al., 2011). Ceramide, a sphingolipid metabolite, serves as a strong autophagy activator (Scarlatti et al., 2004). Inhibiting synthesis of inositol phosphorylceramide reduces autophagy (Yamagata et al., 2011). Mitophagy, the degradation of mitochondria via selective autophagy, is linked to the phospholipid biosynthesis pathway for the conversion of PE to PC by the two methyltransferases, EBP cholestenol delta-isomerase (EBP, also known as CHO2) and phosphatidylethanolamine *N*-methyltransferase (PEMT) (Sakakibara et al., 2015). In addition, the autophagic digestion of LDs through lipophagy in liver is an essential process to obtain energy (Cai et al., 2016). Thus, the composition of membrane lipid seems to be a hallmark of autophagy induction.

## Lipid Peroxidation and Autophagy

Cell death has multiple forms, each exhibiting different molecular mechanisms and signal transductions (Tang et al., 2019). Although autophagy generally promotes cell survival through removing damaged organelles and oxidized molecules, it can also cause cell death under certain circumstances. This type of regulated cell death requires autophagy machinery and is termed as autophagy-dependent cell death by the Nomenclature Committee on Cell Death (Galluzzi et al., 2018).

Lipid peroxidation is a chain reaction of the oxidative degradation of lipids. In the reaction, an initiator radical first takes an allylic hydrogen of the unsaturated lipid and generates a corresponding radical. The free radical then reacts with an oxygen molecule to generate a corresponding peroxy radical, which captures the allyl hydrogen of another molecule and converts it into a hydroperoxide. Polyunsaturated fatty acids (PUFAs) are susceptible to peroxidation to yield various degradation products, such as malondialdehyde (MDA) and 4-hydroxy-2′-nonenal (4HNE) (Ye et al., 2016). These lipid peroxidation products influence cell fate partly through the activation of autophagy. For example, 4HNE can induce autophagy through the activation of c-Jun amino-terminal kinase (JNK) (Csala et al., 2015). The activation of JNK is accompanied by BCL2 being dissociated from BECN1 or by the induction of heme oxygenase 1 (HMOX1, also known as HO1) expression and MAP1LC3-II formation (Velez et al., 2011; Haberzettl and Hill, 2013). Other signaling associated with 4HNE-induced autophagy are the MAPK, mTOR, and protein kinase C pathways (Martinez-Useros and Garcia-Foncillas, 2016). In addition to inducing autophagy at lower concentrations, 4HNE can inhibit autophagic flux at higher concentrations (Dodson et al., 2017), indicating a negative feedback mechanism to limit excessive activation of autophagy during lipid peroxidation.

Lipid peroxidation is implicated in various kinds of regulated cell death (Kang et al., 2018; Su et al., 2019). In particular, increased lipid peroxidation is an important signal for triggering ferroptosis, an iron-dependent form of cell death that was first identified in mutated RAS cancer cells (Dixon et al., 2012). The molecular mechanism of ferroptosis is complicated, depending on the context (Xie et al., 2016; Stockwell et al., 2017; Dai et al., 2020a). There are many connections between lipid metabolism and ferroptosis. Lipid biosynthesis that depends on acyl-CoA synthetase long-chain family member 4 (ACSL4) (Yuan et al., 2016; Kagan et al., 2017) and subsequent lipoxygenase-dependent lipid (e.g., PUFAs) peroxidation (Yang et al., 2016) promotes membrane rupture during ferroptosis. NADPH oxidases (NOXs) and other oxidases may also facilitate membrane oxidative injury during ferroptosis (Gaschler and Stockwell, 2017; Xie et al., 2017). In contrast, several antioxidant or membrane repair mechanisms can prevent ferroptosis. The main anti-ferroptosis mechanisms include system xc^–^-mediated glutathione peroxidase 4 (GPX4) activation (Dixon et al., 2012; Yang et al., 2014), apoptosis-inducing factor mitochondria-associated 2 (AIFM2)-mediated coenzyme Q10 production (Bersuker et al., 2019; Doll et al., 2019), endosomal sorting complexes required for transport (ESCRT)-III–mediated membrane repair (Dai et al., 2020c, d), and nuclear factor, erythroid 2-like 2 (NFE2L2, also known as NRF2)-mediated antioxidant response (Sun et al., 2016a, b; Dodson et al., 2019).

**FIGURE 3:**
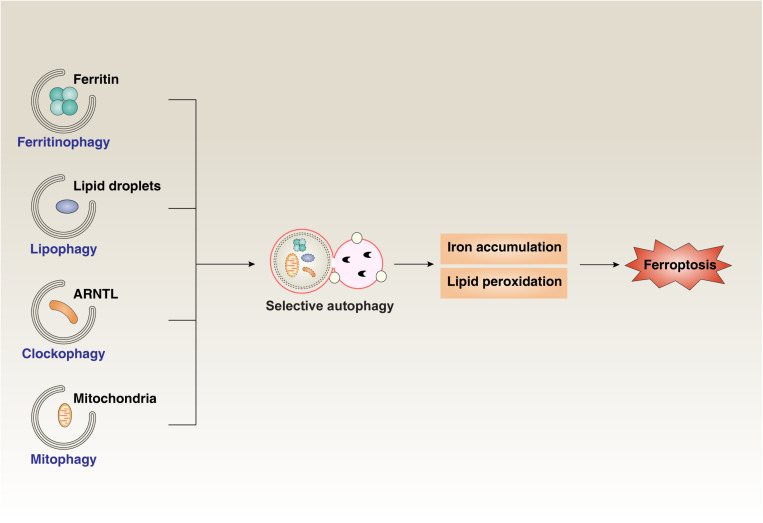
The role of selective autophagy in ferroptosis. Ferritinophagy, clockophagy, lipophagy, and mitophagy promote the degradation of the iron-storing protein ferritin, the core circadian clock protein ARNTL, lipid droplets, and mitochondria, respectively. Activating these types of selective autophagy results in iron accumulation and lipid peroxidation, which finally induces ferroptotic cell death.

Early studies indicate that ferroptosis is different from other forms of regulated cell death, such as apoptosis, necroptosis, and autophagy (Dixon et al., 2012). However, increasing studies suggest that ferroptosis exhibits a particular relationship with autophagy during anticancer therapies, tumorigenesis, inflammatory injury, and tissue fibrosis (Kang and Tang, 2017; Zhou et al., 2019; Liu et al., 2020) (Figure 3). Several types of selective autophagy, such as ferritinophagy, clockophagy, lipophagy, and mitophagy, promote ferroptotic cell death through degradation of the iron-storing protein ferritin, the core circadian clock protein aryl hydrocarbon receptor nuclear translocator-like (ARNTL, also known as BMAL1), LDs, and mitochondria, respectively (Hou et al., 2016; Basit et al., 2017; Bai et al., 2019; Liu et al., 2019; Yang et al., 2019). CMA also promotes ferroptosis through HSP90-mediated GPX4 degradation (Wu et al., 2019). Moreover, BECN1 facilitates ferroptosis through directly inhibiting SLC7A11/system xc^–^ activity (Song et al., 2018) or inducing ferritinophagy (Zhang et al., 2018). The stimulator of interferon response cGAMP interactor 1 (STING1, also known as TMEM173), an ER-associated protein involved in immunity, infection, and coagulation, connects mitochondrial DNA stress to autophagy-dependent ferroptosis (Li et al., 2020). Nanoparticle ferritin-bound erastin and rapamycin (NFER), a nanodrug, exhibits a robust ability to induce ferroptosis and autophagy to inhibit tumor growth (Li et al., 2019). The release of damage-associated molecular patterns (DAMPs) from ferroptotic cells serves as a mediator implicated in immune cell activation (Wen et al., 2019) and tumorigenesis (Dai et al., 2020b). In addition to cancer biology, autophagy-mediated ferroptosis is also implicated in hepatic fibrosis and neurodegenerative disease (Zhang et al., 2018; Kong et al., 2019). These findings may provide a useful framework for understanding the pathological characteristics of autophagy-mediated ferroptosis in diseases.

## Lipid Metabolism Disorders and Autophagy

Autophagy is tightly regulated by ATG genes. When these genes are mutated, a series of diseases, such as cancer, infectious disease, and neurodegenerative disease, can be induced. In addition, impaired autophagy is also closely related to the pathology of several lipid metabolic disorders discussed below.

Lysosomal storage diseases (LSDs) are a class of genetic disorders in which proteins responsible for digestion or absorption of endocytosed material do not function or localize properly. The resulting cellular “lipid indigestion” or “lipid digestion defects” cause a buildup of intracellular storage that contains unprocessed lipids (Kiselyov and Muallem, 2008). LSDs consist of a group of rare inherited metabolic disorder diseases, such as Niemann-Pick C1 (NPC1) disease, G(M1)-gangliosidosis, Gaucher disease, Danon disease, Pompe disease, mucolipidosis type IV disease, and neuronal ceroid lipofuscinoses (NCLs). Impaired autophagy activity is commonly responsible for these LSDs (Seranova et al., 2017). For example, NCLs can be caused by mutations in lysosomal proteases, which leads to a deficiency in the autophagy-dependent degradation of NCL proteins (Brandenstein et al., 2016). Mutated NPC1 protein can block autophagy induction through the inhibition of SNARE-dependent membrane fusion, whereas ATG5-deficient cells exhibit increased NPC1protein accumulation (Sarkar et al., 2013). Thus, the pharmacological induction of autophagy may ameliorate the phenotypes of LSDs.

Preeclampsia is a pregnancy complication characterized by high blood pressure and signs of multiple organ damage (e.g., liver and kidney). Preeclampsia is associated with increased oxidative stress, which can cause autophagy-dependent cell death in extravillous trophoblasts. Mechanistically, oxidative stress reduces lysosomal activities and enhances *de novo* sphingolipids synthesis, which finally results in ceramide overload-dependent autophagic cell death and subsequent inflammation response (Melland-Smith et al., 2015). In addition to excessive autophagy-mediated cellular damage in extravillous trophoblasts, mild levels of autophagy may promote cell survival under hypoxic and low-nutrient conditions (Nakashima et al., 2017). It remains unknown whether a systemic autophagy response affects pregnant women.

The liver is the hub of fat transport. After fat is digested and absorpted, a portion of it enters the liver, and then it is converted into body fat and stored. The liver is also one of the main organs for the synthesis of FAs, cholesterol, and phospholipids in the body. Excess cholesterol is excreted with bile. Lipid metabolic imbalance leads to lipid accumulation in the liver, resulting from steatosis due to non-alcoholic fatty liver disease (NAFLD). The level of lipids in the liver is modulated by lipophagy, and impaired lysosomal pathways are involved in the pathogenesis of NAFLD. In contrast, the activation of autophagic pathways has been shown to ameliorate steatosis and NAFLD in animal models (Ma et al., 2013; Xiao et al., 2016; Kim et al., 2019). These findings suggest that autophagy activators may have therapeutic potential in NAFLD, which includes a spectrum of hepatic disorders associated with obesity.

Altered lipid metabolism and autophagy also contribute to neurodegenerative diseases, such as Parkinson’s disease (PD), a progressive disorder that affects movement. Specific gene mutations, such for as PTEN-induced kinase 1 (PINK1), increase the risk of PD. PINK1 is an important regulator of mitochondrial quality through multiple mechanisms, including mitophagy (Rub et al., 2017). Depleted or mutated PINK1 can increase mitochondrial oxidative injury, ER stress, and mitophagy deficient, which leads to cell death, inflammation, and immune suppression in various diseases (Kang et al., 2016; Li et al., 2018). Of note, reduced hydrolase activity has shown to increase cholesterol accumulation during PD development (Garcia-Sanz et al., 2017). Thus, reducing lipid storage may restore the activity of autophagy, especially mitophagy, to alleviate mitochondrial damage in PD (Han et al., 2018).

Metabolic syndrome includes a cluster of conditions, such as hypertension, hyperglycemia, excessive waist fat, and abnormal cholesterol levels. Autophagic activity is significantly reduced in metabolic syndrome, which increases the risk of obesity, type 2 diabetes, and atherosclerosis. The inhibition of autophagy promotes lipid accumulation, mitochondria dysfunction, and ER stress (Perrotta and Aquila, 2015; Zhang et al., 2015; Martinez-Useros and Garcia-Foncillas, 2016). In contrast, the activation of autophagy may decrease metabolic syndrome-related diseases.

## Conclusion and Perspective

Autophagy is a conserved adaptive response to environmental changes and plays a pivotal role in cell survival and death. It can degrade aging organelles and proteins to produce amino acids, nucleotides, and FFAs for cell survival. At the same time, it can also be used as an active mechanism to induce autophagy-dependent cell death. Generally, ceramides are involved in pro-survival autophagy, while PUFAs are involved in pro-death autophagy. The process of autophagy is regulated by a series of complex signaling molecules and metabolic pathways. Lipid metabolism plays an important role in regulating multiple cell processes. In the past 10 years, there have been major breakthroughs in understanding the crosstalk between lipid metabolism (e.g., digestion, absorption, catabolism, biosynthesis, and peroxidation) and autophagy. In particular, lipid metabolism has been found to be involved in the formation of membrane structures related to autophagy. Inhibiting TG, CE, and FA or increasing membrane lipid biosynthesis is responsible for the induction of autophagy. Moreover, autophagy promotes lipid catabolism and lipid peroxidation-induced cell death, such as ferroptosis. Targeting the autophagy pathway has received extensive attention in human diseases, including lipid metabolism-related disorders. Although these advances in knowledge have propelled the field forward, there is still much to explore. For example, how does autophagy function in lipid metabolism pathways in different cells or tissues? To what extent does the lipid context around membranes affect autophagy induction? How does autophagy switch from pro-survival mode to a pro-death one that ruptures the membranes? To what degree is selective autophagy specially linked to ferroptotic cell death? Which ATG modifications are responsible for lipid disorder phenotypes? A better understanding of the mechanisms of autophagy and possible links to lipid metabolism will undoubtedly promote potential treatments for a variety of diseases.

## Author Contributions

YX and DT conceived of the topic for this review. All authors listed have made a substantial, direct and intellectual contribution to the work, and approved it for publication.

## Conflict of Interest

The authors declare that the research was conducted in the absence of any commercial or financial relationships that could be construed as a potential conflict of interest.
